# Exploring the Potential of Bioactive Peptides: From Natural Sources to Therapeutics

**DOI:** 10.3390/ijms25031391

**Published:** 2024-01-23

**Authors:** Kruttika Purohit, Narsimha Reddy, Anwar Sunna

**Affiliations:** 1 School of Natural Sciences, Macquarie University, Sydney, NSW 2109, Australia; kruttika.purohit@students.mq.edu.au; 2 Australian Research Council Industrial Transformation Training Centre for Facilitated Advancement of Australia’s Bioactives (FAAB), Sydney, NSW 2109, Australia; n.reddy@westernsydney.edu.au; 3 School of Science, Parramatta Campus, Western Sydney University, Penrith, NSW 2751, Australia; 4 Biomolecular Discovery Research Centre, Macquarie University, Sydney, NSW 2109, Australia

**Keywords:** peptidomics, therapeutic peptides, mass spectrometry, bioactivity assays, structure–activity relationship

## Abstract

Bioactive peptides, specific protein fragments with positive health effects, are gaining traction in drug development for advantages like enhanced penetration, low toxicity, and rapid clearance. This comprehensive review navigates the intricate landscape of peptide science, covering discovery to functional characterization. Beginning with a peptidomic exploration of natural sources, the review emphasizes the search for novel peptides. Extraction approaches, including enzymatic hydrolysis, microbial fermentation, and specialized methods for disulfide-linked peptides, are extensively covered. Mass spectrometric analysis techniques for data acquisition and identification, such as liquid chromatography, capillary electrophoresis, untargeted peptide analysis, and bioinformatics, are thoroughly outlined. The exploration of peptide bioactivity incorporates various methodologies, from in vitro assays to in silico techniques, including advanced approaches like phage display and cell-based assays. The review also discusses the structure–activity relationship in the context of antimicrobial peptides (AMPs), ACE-inhibitory peptides (ACEs), and antioxidative peptides (AOPs). Concluding with key findings and future research directions, this interdisciplinary review serves as a comprehensive reference, offering a holistic understanding of peptides and their potential therapeutic applications.

## 1. Introduction

The significance of peptides as physiologically active and therapeutically beneficial molecules has been increasingly acknowledged in recent years. Peptides have become popular drug candidates in the current drug development pipelines, given their appealing pharmacological profile and inherent properties [1]. The high specificity of peptides has been observed to translate into safe, tolerable, and efficacious therapeutics [1].

The use of peptides for therapeutic applications has evolved over time and continues to advance further with improvements in drug development and therapy approaches [2]. It was roughly a century ago that pioneering therapeutic biomolecules such as the opiate morphine and cyclic peptide penicillin were first used during World War II [3]. Around the same time, in the early 1920s, larger polypeptide insulin isolated from natural sources became prominent [3]. However, these large protein-based molecules have low oral bioavailability and are unsuitable for oral delivery; thus, typically require injections (either via intravenous, intramuscular, or subcutaneous routes). On the contrary, the pharmaceutical industry as a whole has long been attracted to oral therapies as they are easiest to administer and ensure greater patient compliance, in turn leading to an initial neglect of proteinaceous therapies [4]. Moreover, the short half-life of plasma, due to rapid degradation by a range of endogenous proteases, is another factor that tempered the enthusiasm for proteinaceous therapeutics [5].

In recent years, with several modern-day technological breakthroughs, there has been a renewed interest in the usage of peptides, both for diagnostic as well as therapeutic applications [3]. Specifically, the advent of sequence elucidation techniques and the current high-throughput analytical methods with high resolution and sensitivity have facilitated the discovery and molecular characterization of a multitude of novel peptides with therapeutic potential [2,3].

Bioactive peptides are specific small amino acid fragments (generally 3–20 amino acids long) obtained from natural sources, capable of causing physiochemical changes in normal body processes [6]. These physiochemical effects exerted by bioactive peptides result from their unique amino acid composition, sequence, and molecular weight [6]. Since most bodily functions result from amino acid interactions (either as interactions between small peptide fragments or as small protein chains), therapeutic peptides hold great potential for a wide spectrum of applications [7].

Structurally, bioactive peptides occupy a unique gap between the typical spectrum of small chemical-based compounds and larger biopharmaceuticals. They are comparatively small in size and show high oral bioavailability. Thus, bioactive peptides represent a type of molecule with high specificity and potency, and fewer side effects and immunogenicity than those of large protein-based biopharmaceuticals [4]. Moreover, peptide-based therapeutics are associated with easy and less complex production processes than their larger protein-based counterparts [1]. Nonetheless, despite their attractiveness, natural peptides possess some inherent drawbacks, such as weak membrane permeability, metabolic instability, and poor in vivo solubility [8,9]. These factors constrain their therapeutic applications through oral administration. To tackle this challenge, peptide therapeutics are primarily administered through the parenteral route [10]. Moreover, recent innovative approaches are exploring alternative administration routes [10]. Consequently, the development of novel drug delivery systems has also enabled the oral administration of peptides [3].

This review commences with a concise exploration of diverse sources of bioactive peptides, progressing to an examination of key methods employed for their extraction. Subsequently, the review focuses on untargeted mass spectrometric techniques, followed by an in-depth discussion on various in silico and in vitro bioactivity assays, along with structure–activity relationship (SAR) considerations for the functional characterization of peptides. In compiling this review, pertinent studies were sourced through an electronic search encompassing PubMed and Google, spanning the years 1991 to 2023. The search strategy involved the primary keyword “bioactive peptides” coupled with additional terms such as “natural sources”, “extraction approaches”, “MS analysis”, “bioactivity assays”, and “SAR” to identify relevant literature. Notably, the selection of eligible studies prioritized primary sources over review articles. Readers seeking further exploration are directed to recent reviews [8,11,12,13,14,15,16,17] for additional insights and comprehensive understanding.

## 2. Peptide Discovery

### 2.1. Natural Sources

Nature is a rich source of an impressive variety of biologically active peptides, expressed in most living species [3]. Accordingly, organisms represent an excellent first source for peptide discovery and exploration activities.

Peptides within a multicellular organism are vital in human physiology, primarily as signalling agents that regulate physiological processes such as growth, defence and immunity, homeostasis, and others [18]. Over the years, these peptides have evolved to express their inherent bioactivity even outside the source organism. Interestingly, many of these evolutionary processes have been a means of active predation, such as in venomous arthropods [19]. For instance, Exenatide, indicated for treating type II diabetes, is a synthetic analogue derived from the glucagon-like peptide 1 (GLP-1) agonist found originally in the salival hormone of the Gila monster lizard [19]. While the venomous effect of this GLP-1 agonist is due to disruption of neurotransmission, low to moderate doses of such peptides can counteract neurological or cardiovascular disturbances or even be employed to alleviate pain [3].

Another rich resource for bioactive peptides is marine organisms. Marine life is constantly exposed to drastically hostile environments, full of microbes that might infect an organism at every opportunity. As a result, most marine organisms, notably sponges, molluscs, ascidians, and a few seaweed species, are known to be producers of antimicrobial peptides that can be employed for human use [18]. Lantibiotics, with a characteristic thioether linkage; the N-acyl moiety containing cytotoxic peptide sourced from the sponge *Theonella swinhoei*; and cytotoxic cyanobactins with heterocyclic thiazole and oxazole moieties obtained from tunicate *Lissoclinum patella* are a few examples of ribosomally synthesised modified peptides [18,20]. Whereas, Vancomycin, pristinamycin, cyclosporine A, and actinomycin D are a few prominent examples of therapeutic non-ribosomally synthesized peptides derived from marine sources [21].

Plants represent another rich resource for natural therapeutically active peptides, with proteins as their second most abundant component (20%). Plant peptides have long been investigated for their potential neutraceutical and pharmaceutical applications [22]. Peptides have been extracted from legumes such as soybeans (source for anticancer RGD polypeptide, lunasin), edible rhizomes such as ginger (source for antihypertensive VTYM, anticancer RALGWSCL, antioxidant, and antimicrobial peptides), and turmeric (source for antioxidant peptide WTLTPLTPA, and anti-ACE peptides CACGGV, DVDP, and CGVGAA), peas, and lentils [23,24,25]. Similarly, various dietary dipeptides and tripeptides that have a significant impact in vivo (angiotensin-converting enzyme (ACE) inhibitors, antioxidative, immunomodulatory, etc.) are also found in fermented milk and dairy products such as yoghurt and cheese [20].

Bioactive peptides from natural sources have undergone years of evolutionary chemical and structural modifications and, hence, have considerable advantages over chemically synthesized peptide analogues. These advantages, which include high stability, high target affinity, and fewer off-target side effects, are particularly important when prolonged/long-term duration of treatment is required for chronic conditions [3,26]. Although not exhaustive, Table 1 lists some of the classical bioactive peptides from natural sources with their potential therapeutic usages. These peptides are either naturally expressed in the source organism or are generated by enzymatic hydrolysis of plant/animal proteins.

### 2.2. Peptidomics and Search for Novel Peptides

One can only begin to fathom the magnitude and seemingly endless varieties of peptides present in nature. Many of these peptides show considerable therapeutic and diagnostic potential, thus completely justifying the increased emphasis on studying peptides and their bioactivities. Schrader [47] defines this study of peptides present in a biological sample, i.e., the peptidome, as the science of ‘peptidomics’ (the term was first used in 2001), in analogy to ‘proteomics’ [48].

Endogenous peptides in vivo are primarily formed due to physiological enzymatic or non-enzymatic breakdown, gene-encoding (such as in the case of bioactive peptides, e.g., neuropeptides), and other enzymatic gene-independent processes [49]. Bioactive peptides, in general, are inactive within their regular parent protein, i.e., propeptide conformation [26]. When cleaved by proteolytic enzymes, these protein molecules generate a bioactive peptide fragment [50]. One such example is the endogenous conversion of proinsulin to active insulin. Gene-encoded peptides or short open reading frame (ORF)-encoded peptides or SEPs (i.e., bioactive peptides) are another form of endogenous peptide [51,52]. These peptides are generally unstable, typically weakly translated, and not secreted in vivo, resulting in low copy numbers [53].

Peptidomics chiefly employs high-performance liquid chromatography (HPLC) and mass spectrometry (MS) approaches to extensively analyse endogenous/native peptides (expressed peptides along with their post-translational modifications) even without prior knowledge about their bioactivities. This technology is tightly linked and is driven by the advancements in MS, and bioinformatics, essential to record and even cope with the massive amount of datasets generated [3].

**Figure 1:**
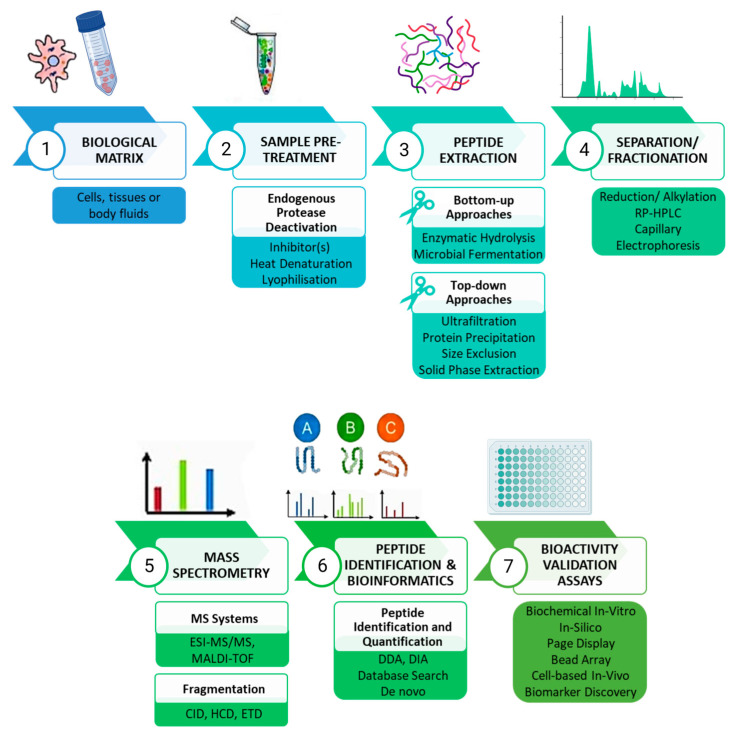
A representative peptidomics-based workflow for isolation and characterization of novel bioactive peptides from natural sources. Created with BioRender.com (accessed on 12 December 2023).

The generic peptidomics analytical workflow starts with the sample pre-treatment phase, followed by peptide extraction, separation, or fractionation to purify the extract, followed by MS analysis and subsequent peptide identification and quantification (Figure 1).

## 3. Peptide Extraction Approaches

The main prerequisite for peptidome analysis is the isolation of peptides from the biological matrices into a suitable solvent that is fit for liquid-chromatography-based MS (LC-MS) analysis. However, since these bioactive peptides within any given biological matrix are present in very low concentrations, all the analytical and sample preparation techniques used need to be highly sensitive but adequately generic to detect as many peptides as possible and, at the same time, remove all other highly abundant and interfering biomolecules such as proteins or lipids [47,50]. The peptidome in itself is also extraordinarily diverse, with peptides varying in their size, polarity, and/or solubility. Furthermore, as López-Otín and Matrisian [5] point out, biological matrices also show the presence of numerous endogenous peptidases. Thus, the peptide extraction process should be quick enough to avoid regular enzymatic degradation [47].

Immunoassays such as ELISA, which can selectively extract specific peptides from heterogeneous matrices using antibodies, are most employed for targeted peptidomics [50]. Immunoaffinity peptide enrichment approaches can also be used in a separation step before LC-MS to purify peptides of interest and improve the assay’s sensitivity to detect low-abundance peptides [54]. Lee et al. [55] effectively utilized this approach with antibody-coupled beads for multiplexing quantitation of gut hormones. Although the approach is highly specific and sensitive, effectively quantitating peptides, the method is not much used for analysing global peptidomes. For discovery-based global peptidomics, the extraction method ought to be fairly generic to capture the maximum possible number of peptides while removing the unwanted interfering proteins present in high abundance [50]. The only exception to this would be using antibodies to isolate a particular cell type using their surface antigens, thus enabling global peptidomic studies for a subset of the original complex matrix [56]. This review focuses on untargeted novel peptide discovery and only covers peptide extraction approaches concerning the global peptidome rather than targeted peptide extractions.

### 3.1. Bottom-Up Approaches

For years, bioactive peptides from food-based sources have been discovered using the classical/bottom-up approach (Table 2). This approach involves hydrolysing the proteins with food-grade enzymes or, alternatively, bacterial fermentation to release several peptide fragments in the hydrolysate [12]. For the peptide hydrolysates thus obtained there is then an extensive database search to identify the peptide fragments followed by in vitro testing to determine their biological activity, and, if shown to have good activity, they are developed further as a therapeutic.

Enzymatic hydrolysis and microbial fermentation methods are the most efficient biological peptide synthesis approaches in terms of high oral bioavailability, fewer adverse effects, and ensuring overall high efficacy, safety, and protease resistance. Nonetheless, the microbial enzymes used in these processes can cause concern regarding the immunogenicity or presence of biological impurities [26]. Commercially purified enzymes are thus employed to minimize impurities and allow for better control over the reaction.

#### 3.1.1. Enzymatic Hydrolysis

Protease-catalysed enzymatic hydrolysis involves cleaving the peptide bond to yield numerous peptide fragments. This is by far the most common, widely used strategy for generating bioactive peptides. Proteases cleave proteins at specific sites and are broadly classified as endopeptidases (serine-, aspartic-, cysteine-, and metalloproteases) and exopeptidases (aminopeptidases and carboxypeptidases) [61]. ACE-inhibitory peptides and calcium-binding peptides are examples of two of the most frequently produced bioactive peptides using tryptic enzymes [25,62,63,64]. Similarly, various other enzymes, such as pepsin, papain, alcalase, bromelain, α-chymotrypsin, and neutrase, with known cleavage sites, have also been successfully utilized for the enzymatic hydrolysis of proteins [41,65].

In a variation, **ultrasonic-assisted protein hydrolysis** has also been explored to facilitate the production of peptides with low molecular weight [66]. Using a suitable enzyme(s) with high specificity and having adequate control over the process parameters is the key to producing peptide hydrolysates with the required biological and techno-functional properties such as size, quantity, amino acid composition, and sequence of the peptides [67,68].

In our view, enzymatic hydrolysis emerges as the most suitable and convenient method for exploratory studies focused on producing large quantities of bioactive peptides from food proteins. Nevertheless, a significant consideration with this approach is that it results in a mixture of peptides with varying molecular weights and peptide sequences. Consequently, a comprehensive separation and purification step is necessary, making the process more laborious and time-consuming.

#### 3.1.2. Microbial Fermentation

Microbial fermentation is a less preferred extraction approach than enzymatic hydrolysis due to longer reaction times, issues with the scalability of operations, and unpredictability of the peptides produced [12]. This method involves culturing a specific known bacteria, yeast, or even filamentous fungi [69] on proteinaceous substrates. The growing microbes, in turn, release their proteolytic enzymes in the matrix as they grow, resulting in protein hydrolysis to yield peptide fragments. The bacterial and/or fungal strain(s), the protein substrates used, and the fermentation time and process parameters dictate the hydrolysis extent, allowing for manipulating the peptide formation process [12,70,71].

The lactic acid bacteria proteolytic system used with different proteinaceous substrates such as fish, soy, and dairy products like yoghurt, cheese, and sour milk has led to the development of a variety of ACE-inhibitory, antimicrobial, antioxidant, and immunomodulatory bioactive peptides [62,71,72].

However, the protein hydrolysates obtained using this method are known to show different functionalities owing to the differences in the proteolytic systems possessed by different microbes. This was confirmed by El-Fatteh et al. [73] in their experiment wherein they studied 14 commercially available dairy starters for their differences in proteolytic activity and the bioactivity of the peptides (ACE-inhibitory and antioxidant) produced after milk fermentation. The unpredictability in the peptides obtained is also highlighted when the same bacterial species differing in their proteolytic capabilities yield different peptides [12]. For example, Sanjukta et al. [74] showed that two sets of soybean protein matrices fermented with *Bacillus subtilis* MTCC5480 and *B. subtilis* MTCC1747 resulted in varying degrees of hydrolysis and of free amino acids being generated.

### 3.2. Top-Down Approaches

Top-down approaches differ from those mentioned earlier in that they do not use proteolytic enzymes (Table 2). The use of proteases often leads to losing critical information about intact peptides and/or truncated proteins from the sample, making top-down approaches a preferred choice. Endogenous peptides or truncated protein molecules in a biological matrix are directly subjected to ionization and fragmentation, followed by mass spectrometric analysis [75]. This approach thus allows for complete characterization of the peptide in its primary conformation.

#### 3.2.1. Ultrafiltration

Centrifugal ultrafiltration is a fast, simple, and yet inexpensive method using both centrifugation and molecular-weight cut-off semipermeable membranes to separate low-molecular-weight peptides from high-molecular-weight biomolecules and can be used to identify many novel peptides in serum and plasma [57,58]. Low-molecular-weight peptides, especially from human plasma, exhibit desirable characteristics of biomarkers or drug targets [75]. An example of the use of this extraction technique has been shown for the separation of neuropeptides from a set of cerebrospinal fluid (CSF) proteins [76].

However, a significant shortcoming of using this approach is that peptides with a molecular weight close to the cut-off value, such as some apolipoprotein or lipophilic peptides, might be lost due to their binding with the membrane [50,75]. Moreover, the possibility of filtrate contamination with other biomolecules in the same size range can also not be dismissed.

#### 3.2.2. Protein Precipitation

Protein precipitation is a simple, reproducible, and inexpensive method that effectively extracts peptides from a biological matrix [50,75]. The addition of solvent (organic solvent or acid) causes the precipitation of larger proteins, leaving peptides and small protein fragments in the solution [50,77].

**Organic solvent precipitation** has been previously used to separate low-abundance plasma peptides in a hugely generic process [78]. Acetonitrile is the most efficient organic acid used for this process. Alternatively, ice-cold acetone [59] or methanol can be used, although with lesser efficiency. Even though the process is straightforward, the precipitating solvent chosen must be specific to the biological matrix used to ensure maximum peptide extraction [79]. Organic precipitation is an efficient process, yet a significant risk factor is the entrapment of smaller peptides within large protein aggregates, leading to inconsistent results. To counter this effect, the use of a 100% pure organic solvent is not preferred. Instead, 20–25% water is added to the solvent to enhance the recovery of smaller peptides, even at picomolar concentrations [77].

**Acid-based precipitation** using acids such as ammonium sulphate and ion-pairing reagents such as trichloroacetic acid offers an excellent alternative to organic solvents [59]. The advantage of acidic solvents is the rapid protein precipitation process, ensuring that protease-related degradation does not occur [50].

#### 3.2.3. Size Exclusion Chromatography (SEC)

Size exclusion chromatography (SEC) is another easy analytical approach for extracting low-molecular-weight peptides from complex biological matrices. The chromatographic adsorbent column consists of porous beads with specific pore sizes, such that it excludes the undesired high-molecular-weight biomolecules [49]. In contrast, low-molecular-weight peptides and small proteins enter the beads and are retained, showing delayed elution. However, a downside of SEC is the high elution volume, leading to sample dilution and increased cost when performing sequential analysis using multiple columns (making it low throughput) [50], and the requirement for a higher sample quantity [59]. Another drawback of SEC is its low resolving power, which can be rectified by coupling it with other separation techniques. A combination of SEC with a secondary reverse-phase chromatography column allows for a 2D separation approach [50,59]. In another approach, Tian et al. [80] used mesoporous silica modified with strong cation-exchange and anion-exchange resins for peptidome enrichment.

#### 3.2.4. Solid-Phase Extraction (SPE)

Solid-phase extraction (SPE) is currently one of the most favoured sample preparation techniques, providing a viable solvent-free alternative to liquid–liquid extraction with a large organic solvent requirement. The SPE sorbent (stationary phase) interacts and retains peptides of interest while removing undesired interfering molecules and salts. Different types of SPE sorbents are available and used across high-throughput formats such as multiwell plates and microfibres, the most common are based on hydrophobic interactions [81]. However, newer versions, such as mixed-mode or restricted access matrix sorbents, can be used to obtain exceptionally sensitive extractions, often with a specific subset of enriched peptides [50]. These newer versions are excellent choices for targeted peptide extractions. However, they must be avoided or used cautiously when untargeted full-scale peptidome extraction is desired.

Overall, despite a slight protein co-elution tendency, SPE is a better extraction approach than ultrafiltration, protein precipitation, or SEC [60]. An alternative to avoid this possible issue is combining the benefits of solvent precipitation and SPE. Preliminary extraction with solvent precipitation removes high-abundance proteins from the sample, while the subsequent SPE removes the leftover small hydrophobic molecules [50]. Kay et al. [77] employed this combination strategy to extract and identify peptides derived from pancreatic α-cells and other biomarkers for neuroendocrine tumours.

### 3.3. Peptides with Disulphide Linkages

Disulphide bonds occur in numerous bioactive peptides with cysteine residues such as insulin, somatostatin, vasopressin, and cyclotides as a peculiar, knotted disulphide structural motif [49]. However, not all the currently available standard commercial bioinformatics platforms can accurately match these disulphide-bond-containing peptides against a database [50]. The reduction and alkylation step prior to MS ensures the separation and linearization of peptides, leading to better fragmentation outcomes in tandem MS and, hence, an increased sample coverage, thus aiding in identifying novel peptides [82]. This step is significant in untargeted peptidomics experiments seeking global sample coverage and can be skipped in targeted peptidomics. In targeted studies, disulphide-bonded peptide chains can be particularly tracked based on their *m*/*z* values. For instance, a good example is an MS-based insulin study wherein the insulin B chain subjected to reduction and alkylation was used as a target instead of intact insulin [83].

## 4. Mass Spectrometric Analysis

Mass spectrometry (MS) can analyse simple peptide fragments without requiring any significant manipulations to the sample, merely by incorporating the sample on a MALDI target plate and subjecting it to a matrix solution or by solvent spray extraction, as in the case of electrospray ionization (ESI) [48]. When comparing between MALDI and ESI, the latter allows for higher instrumental flexibility, and thus, can be easily combined with LC systems and/or various mass analysers [84]. Either of these techniques can be used to generate both positive and negative ions. However, positive ionization [M + H+] is preferred for peptide analysis since the positive charge on the peptide can be stabilized by the basic amino acid residues, which are protonated under positive ionization conditions [50,85]. For instance, Nwosu et al. [86] employed the negative ionization approach, complementary to positive ionization, for enhanced detection of N- and O-linked glycopeptides from bovine lactoferrin. In another instance, Penanes et al. [87] demonstrated the protein identification efficiency in negative ionization mode using LysC, Trypsin, AspN, and GluC, hydrolysed peptide. The study with a 1% FDR successfully identified over 100 proteins in human cells.

On the other hand, though MALDI-MS is a powerful peptide analysis tool able to determine spatial protein/peptide structures without a label [88], it cannot be easily coupled to an LC system, thus exhibiting its limitations in measuring peptides from fluid matrices [50,84]. In addition, when the two ionization techniques were employed to analyse low-molecular-weight pancreatic proteins, Nadler et al. [84] observed a significant ion-source-dependent bias in the results. The frequency distributions of several physicochemical properties, such as hydrophobicity and other charge-related properties, were altered owing to the differences between ESI/MS and MALDI/MS. Moreover, post-translation modifications were detected better with the ESI/MS system, implying that this technique is preferable for peptidomic studies [50].

Furthermore, complex heterogeneous biological matrices (body fluids such as blood, plasma, CSF, or tissue samples) make the peptide analysis difficult, and thus, require an initial separation/fractionation step before being subjected to ionization for MS analysis [48,50]. Additionally, sample preconcentration may be necessary for instances where the sample contains a very low concentration of peptides [48]. Better resolution of complex sample mixtures is generally obtained by running mass analysers in tandem.

### 4.1. Separation and Fractionation of Peptide Mixtures

#### 4.1.1. Liquid Chromatography

Liquid chromatography in tandem with high-resolution MS (LC-MS/MS) with 1D HPLC-based separation is usually the conventional way to analyse endogenous peptides in a sample. Although this approach is sufficiently precise, reproducible, and sensitive [50], the use of a reversed-phase (RP) or strong cation-exchange (SCX) column for 2D separation can further improve the resolving power [49].

The necessity of detecting less-abundant peptides in a complex biological matrix to find novel peptides, coupled with the advancements in analytical automation, has driven the development of ultra-low and nano-flow LC systems [48,89]. These systems, in turn, can be connected to nano-spray sources for MS. Commercially available nano-flow separation systems with a flow rate of approximately a few 100 nL/min increase the analyte concentration in the electrospray source despite introducing an equivalent amount of analyte. This contrasts with conventional LC systems (flow rate ~100 µL/min) and is particularly advantageous when analysing complex or highly diluted sample mixtures with a greater incidence of co-elution [50]. This technique of varying the flow rate, allowing for better resolution of peptides, is known as ‘peak parking’ [48]. A low flow rate, although making the process more time-consuming, slows down the elution peaks, increasing the time for mass analysis, and thus, fragmenting more peptides or a specific less-abundant peptide for a longer time. On the other hand, higher flow rates result in sharper chromatographic peaks but can tend to reduce sensitivity [50,90]. Thus, it is essential to strike a compromise between sensitivity and run time, depending on the requirements of the study.

Nano-flow systems are routinely preferred for untargeted peptidomic studies. This is because the long cycle times of data acquisition scans do not influence the peptide data points on the mass spectrum and, at the same time, achieve high-quality MS/MS spectra for database search [50]. Alternatives to liquid chromatography, such as capillary electrophoresis [91] or MALDI, exist [92]. However, the current widespread use of LC-MS systems for proteomic studies makes these alternative approaches less common.

#### 4.1.2. Capillary Electrophoresis

Capillary electrophoresis when used with MS (CE-MS) is an orthogonal separation mode used complementarily with LC-MS for an increased rate of peptide biomarker sequence identification [49,93]. While LC-MS/MS shows a higher count of identified peptide sequences per analytical cycle [93], CE-MS/MS is a highly reproducible approach for identifying the small-sized and highly charged peptides left undetected in the LC-MS approach [91].

Compared with other separation approaches, the primary advantage of CE is its high-resolution separation at a relatively short cycle time and its low sample requirement [91]. Although the low sample volume requirement implies that the sample needs to be highly concentrated, thus making preconcentration necessary for a few samples, this could be beneficial when analysing volume-restricted or scarce biological samples [94]. CE’s other benefits over LC are the robustness of the process and its faster reconditioning ability at varying pH. Another advantage of CE, in regard to MS interfacing, is the use of a buffer system capable of retaining the sample composition throughout the analysis, ensuring a stable ionization environment for the sample compound [91].

### 4.2. Untargeted Peptide Analysis and Bioinformatics

Untargeted peptide analysis studies aim to identify all the peptides present in a sample to assess differential peptide expression between discrete biological groups. Although MS scan functions primarily employ a data-dependent acquisition (DDA) mode workflow, the data-independent acquisition (DIA) approach is slowly gathering significant attention, even though considered more technically challenging.

#### 4.2.1. Data Acquisition

The DDA approach involves, first, analysing precursor ions relative to their retention time in the first mass analyser (MS1). MS/MS spectra of selected *m*/*z* characteristics are then collected, usually corresponding to the high-intensity peaks in the MS1 scan, to fragment this ion further and analyse its constituent fragments using a second mass analyser (MS2). The resultant information is used to conduct database searches and identify the peptides/proteins present in the biological sample. However, despite generating a high count of identifications, the approach is not very objective for analysing highly abundant peptides [50]. Moreover, the approach displays some significant disadvantages of consistency and accuracy in quantification and reproducibility in identification across sample measurements, arising from the randomness with which the instrument chooses precursors for fragmentation.

Short endogenous peptide analysis represents yet another analytical challenge with DDA due to their low abundance in the sample and low ESI efficiency [95]. As a result, an untargeted metabolomics approach is commonly utilized to identify short endogenous peptides, albeit with limitations on deep identification due to the lack of an extensive metabolomics database [96]. Lately, a newer untargeted metabolomics approach for analysing short endogenous peptides has been developed using suspect screening. Suspect screening is a valuable method in cases when analytical standards are unavailable but where a list of all potential targets can be compiled. Since the 20 amino acids can only make up a limited number of possible short peptide sequence (≤4 amino acids) combinations, all the combinations are estimated with their precise masses and used as a reference database to match precursor masses from the experimental spectra for DIA. The fragmented product ions from the experimental spectra are then compared to the product ions generated in silico, thus yielding a corresponding probable peptide sequence and precursor masses [95,96].

In contrast, the DIA workflow (substitute for DDA, not involving random precursor selection) involves precursor-ion selection for fragmentation in an unbiased multiplexed manner based on deterministic duty cycles, producing a composite mixture of MS2 spectra from more than a single precursor ion. The signal, based on its retention time, must then be deconvoluted to match against the corresponding precursor and product ions [50].

Lin et al. [97] employed the streamlined DIA workflow to analyse the serum peptidome of clear-cell renal carcinoma (ccRCC) compared to the DDA approach. The two approaches were compared based on their peptide detection number, reproducibility in peptide identification, and quantitative consistency and accuracy. The DIA approach led to the quantification of approximately double the number of peptides while resulting in just half the variation detected by DDA. And though the total number of peptides identified was comparable over a set of three replicates across both the approaches, the DIA results were more reproducible and accurate.

#### 4.2.2. Peptide Identification

Peptide identification by **database search** works by matching tandem/experimental spectra to the theoretical spectra derived from the peptide library’s known peptides. While shorter peptides (≤4 amino acids) can be identified by suspect screening, regular peptides (≥5 amino acids) employ protein sequence databases such as X!Tandem, SEQUEST, and Mascot, housing thousands of specific sequences [98]. Moreover, structural peptide databases such as StrPep provide valuable insights about the conserved sequences, thus providing a rationale for in silico studies. Since most biopeptide sequences exhibit common structural motifs and appear in similar protein-fold regions, large-scale bioactive peptide structural mapping using fold-recognition approaches has been trialled to predict the corresponding folds and bioactive peptides present [99].

However, using this method might cause a significant loss of information when identifying peptides from non-annotated proteins and small endogenous peptides. Also, the protein databases for many reference annotated proteins from non-model (organisms not yet sequenced) species are often incomplete, thus hampering the identification of small endogenous peptides. The generation of customized databases, usually based on proteome or transcriptome translation, resolves this issue. The flexibility of customizing databases containing all possible polypeptide sequences, thus, allows for deep identification of all annotated and non-annotated endogenous peptides [49]. Special databases for therapeutics and endogenous peptides, such as SATPdb and SwePep, are also available and are routinely updated by the scientific community [50].

On the contrary, **de novo sequencing** identifies peptides by calculating the mass difference between tandem MS-derived peptide fragments without any limitations of a reference peptide library. Thus, the quality of MS data, particularly fragmentation and mass accuracy, is critical to ensure the optimum performance of de novo sequencing. De novo peptide identification is a promising approach to sequence peptides with unknown modifications (particularly endogenous peptides) [100], peptide isoforms, or atypically shaped peptides like cyclotides [49]. Knickelbine et al. [101] employed the de novo sequencing approach for identifying bioactive neuropeptides in the nematode *Ascaris suum*, identifying 9 novel peptides from a total of 12 neuropeptides detected.

However, de novo sequencing is seldom used as the sole approach; instead, it is often combined with the database search approach to minimize errors and achieve comprehensive peptide identification [102]. This is because of its time-consuming nature and the inaccuracies arising from the often ambiguous regions in the output where the precise sequence cannot be determined.

## 5. Peptide Bioactivity

The identified peptides have significant physiological and pathological consequences and need to be mapped further for their functions, biomarker potential (disease-specific or tissue-specific), and enzyme activities. Various assays can be used for this purpose, broadly falling into three categories—biochemical, in silico, and cell-based.

### 5.1. Biochemical In Vitro Assays

Biochemical assays employ cell-free in vitro systems to mimic the biochemistry of a particular physiological process in vivo. Biochemical assays are ordinarily carried out in a competition setup, in which the study compound must displace a known ligand or substrate [103].

In vitro assay systems have been extensively used in characterizing peptides with antioxidant potential, and antihypertensive and anti-adipogenic properties. For instance, Li et al. [104], using Alcalase 2.4L and papain, studied the antioxidant properties of grass carp protein by comparing hydrolysates for the differences in their radical scavenging activity when subjected to a simulated gastrointestinal environment. In another study, Hirai et al. [105] evaluated Activin A for its anti-adipogenic effects by measuring the activity of a glycolytic enzyme, glycerol-3-phosphate dehydrogenase (GPDH). Early-phase Activin A treatment reduced GPDH levels, affected the transcriptional factor cascade, and thereby inhibited adipogenesis.

As discussed above, in vitro assays are commonplace for evaluating peptide bioactivity. However, the results can vary significantly due to the enzyme source, purity, and substrate ratio. Cell culture bioassays, in particular, are complex and often time-consuming, thus leading to results that do not necessarily reflect the true nature of peptides [26]. Hence, newer methods that combine screening approaches with bioinformatic tools, such as phage and bacterial display technologies, are being developed to use disease-specific protein targets and better evaluate the bioactivities of peptides.

#### 5.1.1. Ligand Binding Assays

Ligand-binding-based assays evaluate the direct interaction between the test compound and the desired target entity using radiolabelled moieties and are known to be extremely sensitive as well as robust. The assay estimates the test compound’s ability to inhibit the radiolabelled ligand from binding with the desired target. These assays rely on the principle that endogenous peptides, on binding to a larger protein or macromolecule, exert regulatory functions, and thus, these interacting counterparts of peptides can provide clues for their potential role [49]. However, the assay must be designed to adequately distinguish between the bound and unbound forms of the radioligand [106]. An example of this is the **[^35^S]GTPγS binding assay** to measure the functional consequence of GPCR occupancy by agonists, antagonists, and inverse agonists at its early-receptor-mediated events [107,108]. The assay’s popularity stems from its relative simplicity and generic nature [109], allowing for valuable insights into current pharmacological themes, such as the functions of accessory proteins and peptides in signalling, the inherent activity of receptors, and specific signalling events triggered by receptor agonists [107].

#### 5.1.2. Fluorescence Assays

Recently, fluorescence assays have replaced traditional ligand-based radiolabelled assays due to their advantages of ease of handling, better sensitivity, and flexibility. Fluorescence methods currently employed for high-throughput screening (HTS) include: fluorescence intensity (FLINT), fluorescence resonance energy transfer (FRET), time-resolved fluorescence (TR-FRET), and fluorescence lifetime analysis [103,106].

**FLINT assays**, for instance, monitor the output for a change in total light, and thus, quantify a biochemical reaction or binding events, particularly enzymatic reactions. These assays are further categorized into fluorescence quench assays and fluorogenic assays, based on the fluorescent moieties in a reaction system. While fluorescence quench assays use fluorescently tagged substrates, with the fluorescent tag cleaved during the reaction, the latter measures the formation of fluorescent reaction products [106]. While this assay method is relatively cheap and easy to run, the assay is considerably affected by interfering artifacts such as autofluorescence or the presence of organic fluorophores in the molecules assayed [103,110].

An alternative to FLINT is the use of radiometric fluorescence techniques, which are less sensitive to artifacts. **Fluorescence anisotropy/polarization** is one such popular HTS technology commonly employed in protein–ligand competitive screening. Herein, a fluorophore group is excited with polarized light and the resultant emission is measured. The emission signal is later depolarized as the labelled moiety reduces over time. A fluorophore group binding to a large molecule reduces its mobility, resulting in increased anisotropy, thus allowing the binding to be quantitatively estimated [103].

On the contrary, in FRET, the energy from an excited donor fluorophore is transferred to an acceptor or a quenching group, in turn leading to a measurable emission. Typically, organic fluorescent dyes such as fluorescein or rhodamine are utilized conjugated to the peptide of interest [103,106]. A frequent application of this technique is in protease assays, which involve the uncleaved substrate being quenched to emit fluorescence. However, similar to FLINT, background-fluorescence-related interference is a common problem, often resolved using time-resolved fluorescence instead [106].

**Time-resolved fluorescence** is a variant of FRET that uses lanthanide complexes of europium, samarium, dysprosium, or terbium with long-lived fluorescence (generally 1000 s of µs). Using lanthanide complexes thus eliminates the interference caused by short-lived autofluorescence or background fluorescence [103,106]. Lanthanide chelates have been successfully employed in commercial immunoassays with technologies such as the dissociation-enhanced lanthanide fluoroimmuno assay (DELFIA)—a heterogeneous assay—or homogeneous assays such as LANCE™ (Perkin Elmer Life Sciences) and HTRF^®^ (Cisbio). The DELFIA system is mainly used for cell-based or membrane-based assays due to its broad detection range, high robustness, and sensitivity. However, heterogeneous assays such as DELFIA, involving multiple binding, incubation, and washing steps, were unsuitable for HTS, thus paving the way for developing homogeneous time-resolved fluorescence assays [106]. Both LANCE™ and HTRF^®^ technologies have been applied to detect the bioactivities of enzyme kinase [111] and proteases [112], and several biomarkers like interleukin 1β and TNF-α [113].

### 5.2. In Silico Assays

Function prediction analysis for peptides is performed in silico by combining bioinformatic tools with experimental studies and using available databases for bioactive peptides [49]. These prior computer simulations allow for quick and effective screening of a great number of putative bioactive peptide candidates, which can be examined further for their proposed function with experimental studies. Molecular docking and quantitative structure–activity relationship (QSAR) modelling are the most common in silico approaches [26]. He et al. [114] used the molecular docking approach to confirm the ACE and renin inhibitory (hypotensive) activities of rapeseed protein-derived peptides, while Velarde-Salcedo et al. [115] used the same approach to determine the dipeptidyl-peptidase IV inhibitory activity of peptides obtained from amaranth hydrolysate. In yet another study, Azkargorta et al. [116] performed an in silico predictive analysis to investigate the antimicrobial activity of peptides in the human tear peptidome, later confirmed with in vitro studies.

### 5.3. Phage Display

Phage display technology is a method to identify potential novel therapeutic peptides by an intensive screening of a wide diversity of exogenous peptides displayed on the phage surface, such that peptides binding to the desired target are selected [26,117]. This method is advantageous over other expression systems as it offers high-throughput bio-panning, mimics epitope screening, has a simple preparatory process, and can be easily semi-automated for HTS [118]. Various bacteriophage systems such as λ, T4, T7, and filamentous M13 have been developed; however, filamentous bacteriophages, especially M13, remain the most popular choice.

**Figure 2:**
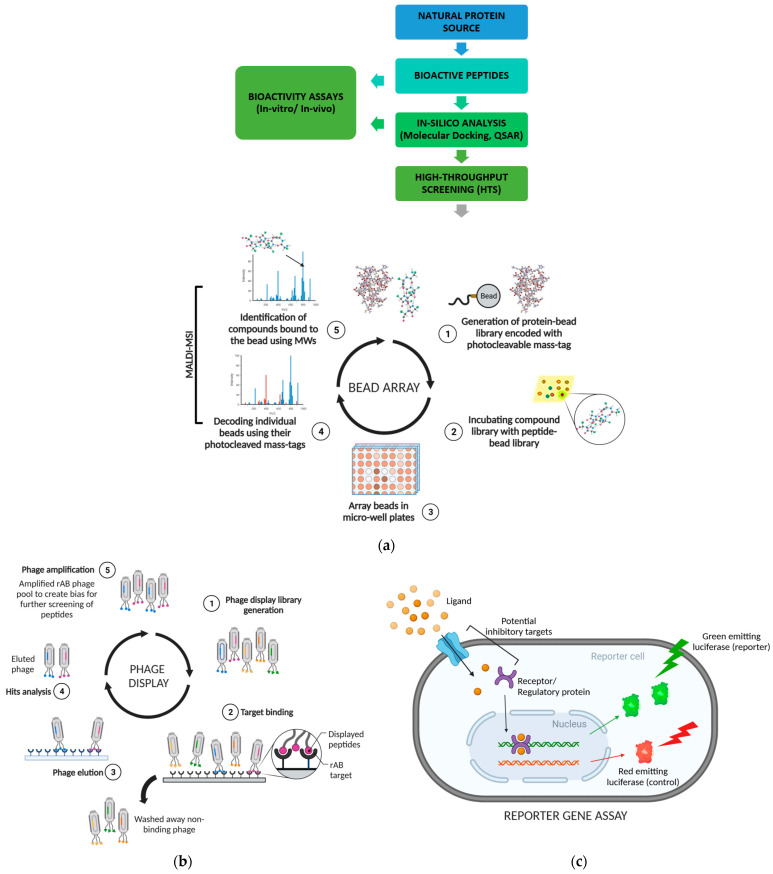
A few of the common screening approaches for bioactive peptides. (**a**) Illustrative representation for bead array to identify peptides based on a protein-bead library with photocleavable mass tags; (**b**) process illustration of phage display involving recombinant antibody (rAB) phage pool for an extensive biased screening of peptides; (**c**) illustrative representation of reporter gene assay measuring gene expression level in response to an interference with cellular signalling pathway. Created with BioRender.com (accessed on 22 January 2024).

For instance, Zani and Moreau [119] used protease-resistant filamentous phages to screen random peptide (with affinity tags) libraries to identify potential protease inhibitors. The resistant phages were separated and amplified after protease exposure to create a bias and further screen peptides for specific purposes (Figure 2a) [117]. Likewise, phage display has been employed to identify several novel tissue inhibitors for the matrix metalloproteinases MMP-1 and MMP-2 [120], and peptide inhibitors for 3-hydroxy-3-methylglutaryl coenzyme A (HMG-CoA) reductase [121] and tyrosinase [122].

### 5.4. Bead Array

Bead-based global proteomic screening (Bead-GPS) is a simple screening approach that involves combining label-free mass spectrometric imaging (MALDI-MSI) with protein microarray technology [123]. The method’s primary advantage is that it facilitates simultaneous screening of several peptides against an entire library. The beads in a high-density random bead array are spatially localized with MALDI-MSI to detect the attached photocleavable mass tags (PC-mass-tag) unique to each peptide/protein. Since each micro-bead can have a distinct protein target and corresponding PC-mass-tag, simultaneous detection of bound compounds is possible using MALDI-MSI (Figure 2b) [124].

This approach has been applied in screening the human protein library for newer autoimmune or anti-tumour agents and biomarkers to be used in immunotherapy or diagnostics and screening peptide libraries against specific targets for initial identification of peptide bioactivities. However, Bead-GPS has been used complementary to and not as a replacement for animal or human studies [123,124].

### 5.5. Cell-Based In Vivo Assays

In contrast to biochemical assays, cell-based assays are more sensitive as they mimic the cellular in vivo context more closely [106]. The three primary scenarios where cell (or organism)-based assays are preferred are (i) the molecular target is unknown; (ii) it is not feasible to satisfactorily reconstitute the desired target in a biochemical assay; or (iii) the desired outcome can only be studied in a cellular context, for instance, cell differentiation, and certain cellular pathways such as signal transduction, membrane transport, metabolism, and cytotoxicity or antimicrobial action [103,106]. Additionally, the functional cell-based assay can adequately distinguish between full or partial agonists and antagonists, inverse agonists, and positive or negative allosteric modulators as well, which is not possible with in vitro biochemical systems [103,106].

Although advantageous in screening multiple targets at once, the major limitations of the cell-based assay are its time-consuming nature with complicated process protocols, a requirement for sophisticated instrumentation, and a general low throughput [106]. Moreover, the non-specific nature and resultant off-target hits often lead to higher target rates, making it mandatory to further screen the primary hits with secondary assays [106].

#### 5.5.1. Cell Viability Assay

A type of cell viability assay used to specifically identify compounds to target cancer cells or pathogens includes monitoring the intracellular concentration changes of ions and other moieties like cAMP or ATP [125]. A range of fluorescent dyes such as Alamar Blue, ruthenium, and other tetrazonium compounds are used, forming reversible complexes with Ca^2+^ or Tl^+^ ions and monitoring their concentration changes. The change in fluorescence intensity upon complex formation indicates cell viability or death [103,106]. Alternatively, changes in Ca^2+^ concentration can also be observed using ion-sensitive Aequorin, a jelly protein emitting strong fluorescence on complexation with Ca^2+^ [106].

The activation/blocking of membrane receptors or ion channels is studied using fluorescent membrane-bound dyes, with varying fluorescence intensity according to the membrane potential [106]. Furthermore, cell death can be monitored by intercalating membrane-impermeable dyes such as propidium iodide [126] into the DNA, staining only the dead cells. Another approach to assess cell viability or death is to measure the intracellular enzymes, such as protease or lactate dehydrogenase (LDH), released into the media [103].

#### 5.5.2. Reporter Gene Assays

Reporter gene assays quantify a eukaryotic cell’s gene expression by transfecting it with a promoter sequence for a reporter gene, whose expression in response to a foreign compound can be easily monitored. The gene expression level varies with whether a compound activates/inhibits the promoter itself or interferes with an entire signalling pathway at any point(s) and is measured by luminescence, fluorescence, or optical readout (Figure 2c) [103,106]. An example is the use of the human H293-NF-κB-RE-luc2P reporter cell line to screen peptides with anti-inflammatory properties [127]. The assay system measures a peptide’s inhibitory ability against TNF-α-induced NF-κB activity.

Significant advantages of reporter assays are their high sensitivity to small transcriptional changes, robustness, and HTS compatibility [26,128]. Moreover, multiple targets can be studied simultaneously by employing different reporters, each constitutively expressed [106,128]. Luciferase (Luc) is the commonly used reporter gene; nevertheless, other reporters such as β-galactosidase (β-gal), chloramphenicol acetyltransferase (CAT), β-lactamase (β-lac), and green fluorescence protein (GFP) are also available. Whilst being widely used, the method is plagued by limitations related to the high variability in cellular response and potential cytotoxicity, mainly due to the long incubation times required for the transcription and translation processes [103,106,128].

A modification of the reporter gene assay, albeit with a better proximal response and a faster response time, is the **secondary messenger assay** that monitors a messenger molecule within a pathway for its expression or transport. The secondary messenger assay is most frequently used for studies involving G-protein-coupled receptor (GPCR) targets. The assay employs calcium-sensitive dyes to study the intracellular Ca^2+^ storage activated by the GPCR target. However, if the target GPCR is not a known Ca^2+^ modulator, it can be manipulated to simulate Ca^2+^ signalling using universal cell adaptors such as promiscuous and chimeric G-proteins [103].

#### 5.5.3. Protein–Protein Interactions

Investigations of protein–protein interactions (PPIs) have suggested their involvement in various molecular mechanisms underlying diseases, making PPIs some of the well-recognized potential therapeutic targets [128,129]. Considerable efforts are underway towards peptide-based innovations to interfere with pathogenic/disease-causing PPIs and modulate downstream signalling. However, such modulations with small chemical molecules are challenging due to the large PPI interfaces. In contrast, the task is much easier with peptides owing to the physiochemical advantages of large and flexible backbones, making them the most suited candidates for PPI inhibitions. Interfering peptides (IPs), i.e., the peptides interfering with PPIs, do so by binding to the large grooves or notches on the PPI interface, thus blocking it [129].

**Protein-fragment complementation assays (PCAs) or biomolecular fluorescence complementation** fuses hypothetical binding partners to two different rationally designed reporter protein fragments (most commonly Luc, GFP, β-gal), enabling formative or inhibitory PPIs detection [103,128]. The split reporter fragments come closer when the bait and prey proteins interact, thus facilitating their non-covalent specific reassembly to their native structure, and a signal is detected [130]. PCAs, being amenable to HTS, have been successfully employed for genome-wide in vivo PPI screening and have also been trialled to examine biochemical networks and screening potential protein inhibitors [128]. Tebo and Gautier [131] have also reported developing a fluorescence-based rapid and reversible complementation system to visualize transient real-time protein–protein interactions in living cells. The system is engineered from the FAST (fluorescence-activating and absorption-shifting tag) fluorogenic reporter, such that it binds explicitly to hydroxybenzylidene rhodanine (HBR) analogues reversibly and facilitates real-time visualization of protein assembly formation and dissociation [131].

The **yeast two-hybrid (2H) assay** is another complementation screening method used to measure protein interactions with other proteins or with DNA. Even though it is widely used for studying the binding of active transcription factors, the approach can be used for screening small molecules that interfere with specific protein–protein or protein–DNA and other drug–target interactions [103,106]. Like PCA, the yeast 2H assay employs the bait–prey interaction system, consisting of a bait protein coupled with a DNA-specific binding domain and a prey protein bound to a transcription activation domain, with a reporter gene inserted after the promoter region. The prey protein, on binding with the bait, brings together the activation domain and the reporter gene in close association, activating it, and thus, enabling detection [103,132]. Activation is prevented in the presence of molecules with inhibitory activity against PPIs [106].

Apart from the conventional transcriptional screening approaches, PPIs can also be examined using other luminescent reporter protein-based high-throughput techniques such as FRET (described earlier) or bioluminescence resonance energy transfer (BRET) [128]. BRET involves using a light-emitting enzyme such as nanoluciferase as a donor, while the acceptor is usually a yellow fluorescent protein [103]. Although BRET assays offer lower intensity when compared to FRET, the technique does not require any excitation light, and thus, translates to minimal autofluorescence or scattering related background signal [133].

#### 5.5.4. Label-Free Detection

Currently, the drug screening process is shifting towards using more physiologically relevant assay systems and departing from the traditional screening methods based on dyes, fluorescent labelling, or overexpressed cell systems, and for GPCRs, avoiding using promiscuous G-proteins. However, a pitfall to this is that endogenous receptors’ expression levels are lower, and thus, require highly sensitive detection methods.

Label-free assays have been devised to study the ligand-induced biochemical response in living cells, most commonly using a biosensor. These assays are not commonplace in primary screening strategies, primarily due to the limited throughput, complexity, and relatively high cost involved, but are employed as confirmatory tests or for the correlations of indirect measurements [103,106]. On the other hand, the assay is more sensitive than other comparable methods, has a relatively short development time, and a single assay can simultaneously study a wide range of cell signalling events, thus making the approach a promising drug screening tool [106,128,134].

#### 5.5.5. High-Throughput Electrophysiology Assays

The recognition of ion channels, specifically voltage-gated ion channels, as potential drug targets, has increased the adaption of impedance-based cellular screening assays for the pharmacological evaluation of therapeutic lead molecules [106,135]. This high-throughput, flexible yet robust assay approach can be used across many applications, from early cell events such as receptor-mediated signal transduction to long-term events like cell proliferation and toxicity [135].

The electrophysiological impedance-based assay is a label-free assay format that works by measuring the ion current flow across a cellular membrane in the presence of a constant voltage, providing important bio-relevant information about therapeutics’ receptor targets in living cells, often in an automated format [103,135]. The technology’s sensitivity to endogenous receptor activation levels in primary cells allows for examining molecular response cascades in their native biological environment, thus creating better cellular models resembling disease states [135].

Automated electrophysiology assays, usually requiring minimal sample volumes, have been particularly developed for studying peptide toxins concerning disease mutation characterization, structure–function studies, or ligand finding. For instance, Chernov-Rogan et al. [136], in a study directed at finding selective blockers for the mutant Nav1.7 channel (sodium ion channel encoded by SCN9A gene), designed an electrophysiology assay based on the Nav1.7 channel’s activator 1KαPMTX peptide, a wasp venom toxin derivative. In another Nav1.7 based study, Xu et al. [137] used high-throughput electrophysiology for analysing the Nav1.7 inhibitory activity of Protoxin-II, a gating-modifier toxin from the Peruvian green velvet tarantula.

### 5.6. AI-Driven Approaches for Peptide Discovery

Analysing bioactive peptides is crucial for the efficient identification and production of peptide-based drugs. However, conventional methods for discovering and manufacturing bioactive peptides are labour-intensive, challenging, and influenced by numerous factors, often necessitating longer experiment times [138]. In response, computational biologists have created various AI tools that use sequence knowledge to predict key peptide features, expediting decision making and exploration, and enhancing data quality for well-defined queries [138]. Numerous data-driven computational methods, particularly artificial intelligence (AI)-based support vector machine (SVM), random forest (RF), extremely randomized trees (ERTs), and deep learning (DL), have been developed to assist in predicting a large pool of therapeutic peptides based on peptide datasets generated by high-performance sequencing [138,139]. For instance, deep learning models for peptide generation such as DeepImmuno-GAN (architecture developed for generating potential MHC-binding peptides) [140], DeepACP and XDeep-AcPEP (for identifying and predicting activities of anticancer peptides) [141,142], ProteinGAN [143], HydrAMP [144], peptide VAE [145], pepGAN [146], and pepVAE [147] have been developed [148].

Additionally, AI-based bioinformatics approaches can also be utilized in 3D peptide structural predictions as an alternative to expensive X-ray crystallography and NMR techniques [138]. Programs such as Pepstr [149], PEP-FOLD [150], and PepLook [151] are optimized for peptides and better suited for peptidomics than protein-centric programs. These tools work by generating several predicted peptide structures de novo, considering the variations in peptide structures [138].

Furthermore, the utilization of deep learning and simulation methods can aid in screening peptides for their bioactivities. Given the numerous similarities between protein–ligand and protein–peptide interactions, employing a hybrid screening strategy can offer valuable insights into efficiently and accurately identifying peptides for a specific target [148]. While publicly available software such as Rosetta (www.rosettacommons.org/software) [152], AutoDock (autodock.scripps.edu), and ZDOCK (zdock.umassmed.edu) [153] exists, only a limited few provide comprehensive and efficient screening pipelines [148].

The AI models, although meant to streamline and make the human analysis process more consistent, come with their own set of limitations. Deep learning prediction models involve a larger number of parameters, posing challenges during training, particularly in instances of insufficient samples or a sparse feature matrix [139]. Despite the increasing number of models, there is a lack of experimental validation to support the reliability of predictions. The sheer volume of data and new information can be overwhelming, thus making it imperative to use AI-based approaches in synergy with biochemical experimental assays rather than relying on them as standalone approaches [138]. In one of the several studies using prediction software, Ding et al. [154] used the AnOxPePred 1.0 (to estimate a free-radical scavenging score and chelation score), AntiInflam, AHTpin, and HemoPI prediction tools, in addition to in vitro assays, to predict the antioxidant, antiinflammtory, antihypertensive, and hemolytic activities of velvet antler blood peptides shortlisted using PeptideRanker based on their potential biological activity scores.

## 6. Structure–Activity Relationship (SAR)

As discussed earlier in this review, numerous bioactive peptides have been isolated from natural sources [155,156]. They include antimicrobial peptides (AMPs) [157,158,159,160], antihypertensive peptides [158,161,162,163,164], antioxidative peptides [164,165,166], immunomodulatory peptides [167,168,169], and anticancer peptides [156,170,171], to name a few. However, proteins and peptides are linear polymers and can also be synthesized as unique combinations of the 20 essential amino acids, each with unique structures.

The backbone structures of different peptides are similar, and they mainly differ in their amino acid sidechains attached to the α-carbon atom (Cα). These biopolymers are formed by peptide bonds (amide bonds) between the adjacent amino acid residues [172]. It is important to determine the primary sequence and the secondary structure of peptides as these structural elements are related to their biological activities. Structural characterization of identified bioactive peptides is usually carried out using the mass spectrometry (MS) technique for amino acid sequencing followed by molecular spectroscopy for secondary structure determination [173,174,175,176]. This review specifically focuses on the structure–activity relationship (SAR) aspects of antimicrobial peptides (AMPs), antihypertensive peptides, and antioxidative peptides (AOPs), given the abundance of literature supporting these prediction models. Other activities, including antiparasitic, anticancer, antidiabetic, etc., are not addressed here due to the limited literature, resulting in inconclusive evidence linking peptide structure to these bioactivities.

### 6.1. Antimicrobial Peptides (AMPs)

Nature provides most life forms with short-cationic antimicrobial peptides (AMPs) as part of their innate immune system [177]. In addition, many food-protein-derived antimicrobial peptides have been discovered too [157,159,160,178,179,180,181,182]. Some examples are IPAVFK, VAGTWY, EQLTK, PYVRYL, and LKKISQ.

The activities of most of these AMPs are linked to their secondary structures [178,180,182,183]. Ref. [183] indicates that the naturally occurring AMPs can be classified into four major categories based on their structure: β-sheet, α-helical, loop, and extended structures. The secondary structures of natural AMPs are predominantly β-sheets and α-helices [183]. However, the literature on the secondary structures of AMPs derived from food proteins is limited [176]. Therefore, limiting the discussion on the SAR of AMPs to natural peptides.

Antimicrobial peptides can also be classified into anionic and cationic peptides based on their net charge [177,184]. In cationic antimicrobial peptides with a net-positive charge, the overall positive charge is contributed to by a significant presence of lysine (K) and arginine (R) residues [177,184,185]. Some cationic AMPs have also been derived from the digestion of food proteins such as milk and egg proteins [182,186,187]. Examples of these peptides are Casosidin-I from αs2-casein [182] and IVSDGNGMNAWVAWR from egg lysozyme [187]. The activity of cationic AMPs has been linked to their ability to bind to the negatively charged outer membrane of Gram-negative bacteria consisting of lipopolysaccharides (LPSs) and causing membrane protrusion leading to bacterial death [188]. Most naturally occurring AMPs are long peptides with more than 30 amino acid residues, except for a few smaller AMPs. In contrast, most food-derived AMPs are smaller peptides having less than 30 amino acid residues. The structure–activity relationship of AMPs, can thus, be summarized as follows:

- α-helix and β-sheet structures are favourable for antimicrobial activity.
- The cationic nature of peptides favours antimicrobial activity.
- Aspartic acid (D)-rich anionic AMPs exist. However, their mode of action is not known.

It is hypothesized that the antimicrobial activity of peptides may be due to several factors, including the amino acid sequence, secondary structure, hydrophobicity, and the net charge. The activity for smaller peptides is likely related to their sequence, hydrophobicity, and net charge. In contrast, secondary structures may play essential roles in addition to the above factors in the case of large peptides. Since information about anionic AMPs is quite limited, this review will focus on the interaction between cationic AMPs and bacterial membranes to understand the mechanism of the antimicrobial action of AMPs.

**Figure 3:**
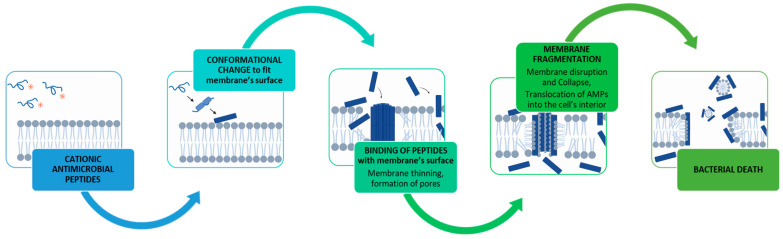
Simplified representation of the mechanism of action of cationic antimicrobial peptides (AMPs) on bacterial cell membrane—cationic AMPs, upon binding to the bacterial cell membrane, cause membrane thinning and disrupt the integrity of the membrane, thus paving the way for the translocation of AMPs into the cell. Created with BioRender.com (accessed on 12 December 2023).

The activity of positively charged AMPs on Gram-negative bacteria is initiated by the electrostatic interaction with the negatively charged lipopolysaccharide cellular membrane (Figure 3). The AMPs may then undergo a conformational phase transition that induces localized thinning of the membrane by forcing polar phospholipid head groups aside and introducing the hydrophobic portion of the peptide into the membrane. As the amount of AMP reaches a threshold concentration necessary for the AMP to enter and traverse the lipid bilayer of the membrane and beyond, the membrane pore will be expanded [189], which is postulated to promote leakage of cytoplasmic components [185] and lead to cell death.

### 6.2. ACE-Inhibitory Peptides (ACEs)

Angiotensin-converting enzyme (ACE) converts angiotensin-I into angiotensin-II, a vasoconstrictor. Overproduction of angiotensin-II is one of the main causes of high blood pressure in patients with hypertension. Suitable ACE inhibitors can block this activity and act as therapeutic agents to control blood pressure in these patients. Several potent ACE inhibitory peptides derived from food protein have been reported in the literature [161,162,163] and, hence, are important candidates for blood pressure control. These inhibitors bind to the C-terminal active site of ACE and block its activity. Peptides that can form multiple contacts with ACE’s active site and establish strong binding are the important candidates.

These food-protein-derived ACEIPs are short peptides with less than ten amino acid residues, except for some naturally occurring longer ACEIPs [190]. Given their short sequences, these peptides are mostly linear, with residues containing multiple binding groups to establish strong binding with ACE [191,192]. Many ACEIPs have some degree of similarity in their primary structures, such as the presence of the amino acids proline (P), tryptophan (W), tyrosine (Y), and arginine (R) in their C-terminal end that have been postulated to contribute to their ACE inhibitory activity [193,194].

A closer look at the specific details of the structure of ACE [191,192] reveals that the active site consists of a zinc coordination site, several hydrogen bonding sub-sites, a proline binding pocket, and a hydrophobic pocket (with “F” residues) with a preference for aromatic amino acids like phenylalanine. It is, thus, imperative for a potent peptide inhibitor to have the following structural characteristics [191,192]:

- Strong chelating groups to efficiently coordinate with the zinc II ion in the active site.
- Suitable functional groups that can form strong hydrogen bonds with appropriate sub-sites in the ACE active site.
- A hydrophobic residue with an aromatic side chain, such as phenylalanine.
- Proline-containing sequences for effective binding.
- Preferably, a short peptide chain with 2–4 residues (a tripeptide may be the best fit).

Di- and tripeptides and their analogues possessing the above structural requirements have displayed extremely high ACE-inhibitory potential [191,192]. These potent inhibitors include (i) CP, CPF, and their structural analogues, such as captopril [192], and (ii) FKP and its structural analogues, like lisinopril [191].

To gain further insight into the structural requirements of ACEIPs, it will be beneficial to consider quantitative structure–activity relationship (QSAR) models [193,194]. These models consider the measured half-maximal inhibition concentration values (IC50) of peptides as the dependent variable and the physicochemical properties of amino acid residues (lipophilicity, steric properties, and electronic properties) as the independent variables that act as predictors.

Some of the ACEIPS predicted by QSAR models include (i) FF, YF, WF, WY, YY, etc., (dipeptides with bulky and hydrophobic side chains); (ii) LKF, LKP, VKP, IRW, LKW, IKP, IKW, etc. (tripeptides with aromatic, positively charged, and hydrophobic amino acids); and (iii) MRFF, MRFP, MFFP, IFFP, IWFP, VWFF, IWFY, VHFY, WIHY, WVWY, WMMY, WMHC, etc., (tetrapeptides and longer peptides) as described by Wu et al. [193,194]. These QSAR models have been developed based on the known active inhibitors, and hence, must be used with caution when interpreting the results of freshly discovered inhibitors [193]. A list of food-derived and natural ACEIPs is provided in Table 3.

From a closer look at the sequences of food-derived and natural ACEIPs, it is evident that most of them contain IPP and VPP fragments in their C-terminal end. Many of these peptides also have other relevant residues (such as F, K, P, W, Y, R), identified based on ACE’s active-site characteristics and the QSAR model. These sequences are indeed observed to be good inhibitors, supporting the predictability of these models. However, these peptides are not designed to make maximum possible contact with all (or most) sub-sites in the ACE active site, and hence, do not form the best inhibitors. Nevertheless, they can be used as lead compounds to construct pharmaceutical-grade ACE inhibitors.

While the food-derived peptides VY (IC50 = 21.88 µM), IY (IC50 = 2.10 µM), and FY (IC50 = 25 µM) are some examples of food-derived dipeptides that essentially fulfil the ACE binding criteria, making them good inhibitors, it is important to note that a tyrosine (Y) residue at the C-terminal end is necessary for these dipeptides. For instance, the peptide YV (IC50 = 575.4 µM) exhibits much lower activity compared to VY (IC50 = 21.88 µM). Likewise, a hydrophobic residue in the N-terminal end enhances the ACE binding efficiency, thus making IY a better inhibitor than VY.

Also, the tripeptides VKP (IC50 = 1.3 µM), IKP (IC50 = 7 µM), and LKP (IC50 = 0.32 µM), all with proline (P) in the C-terminal end, lysine (K) in the middle, and a hydrophobic residue in the N-terminal end, have the potential to make many contacts with ACE’s active-site, and hence, are excellent ACE inhibitors. It is worth noting from the activities of these tripeptides that the increase in hydrophobicity of the N-terminal residue significantly increases the activities (note that L has significantly larger hydrophobicity than V).

Furthermore, the peptides FCVLRP (IC50 = 12.3 µM), IFVPAF (IC50 = 3.4 µM), KPPETV (IC50 = 24.1 µM), and YPYY (IC50 = 90.9 µM) also display good inhibitory activity. It should be noted that the sequences of these peptides contain residues predicted by QSAR studies, and hence, are good ACE inhibitors.

Although many of the above-discussed food peptides display excellent ACE-inhibitory activity, their IC50 values are in the µM range, much below the activities of clinically used ACE inhibitors such as lisinopril (IC50 = 3.72 nM). From these findings, it is hypothesized that a suitable strategy to design a new generation ACE inhibitors should involve (i) the enzymatic production of ACEIPs from food protein, (ii) selecting and using the most active peptides as lead compounds, and (iii) subsequent chemical modification of these lead compounds (peptides) to discover the new-generation ACE inhibitors that fulfil all the structural features for effective binding with ACE.

It should be noted that the active site of ACE is not only responsible for the production of a vasoconstrictor octapeptide (angiotensin II), but also causes the inactivation of a vasodilatory peptide (bradykinin), both mechanisms increasing blood pressure. Therefore, a potent ACE inhibitor is expected to provide double the benefit and is extremely important in treating hypertension, heart failure, myocardial infarction, and diabetic nephropathy [190].

### 6.3. Antioxidative Peptides (AOPs)

The human body produces reactive oxygen species (ROS) and other free radicals during metabolic processes. Consumption of foods rich in antioxidants helps to scavenge these harmful free radicals. Lack of sufficient quantities of antioxidants is one of the key reasons for developing oxidative stress, and prolonged oxidative stress in the body can cause damage to functional biomolecules [208]. Such effects, if untreated, are known to cause several lifestyle illnesses that include, cardiovascular diseases, diabetes, neurological conditions, and cancer [209,210]. Regular consumption of antioxidant-rich foods is, therefore, an important preventative measure to maintain good health.

Food-protein-derived bioactive peptides are known to display high antioxidative activities and help to prevent many lifestyle diseases. The strength of antioxidative agents can be assessed based on their ability to scavenge chemical free radicals (e.g., DPPH, ABTS) and by measuring their ability to chelate with metal ions (e.g., Fe^2+^) to eliminate excessive free ions in the body.

Most naturally occurring antioxidative peptides reported in the literature are larger in size when compared to food-protein-derived peptides. The literature indicates that antioxidant peptides derived from milk and soybean contain 5–16 residues and include hydrophobic amino acids such as proline (P), histidine (H), tyrosine (Y), or tryptophan (W) in the sequences [211,212,213].

To evaluate the quantitative structure–antioxidant activity relationship of peptides, Li et al. [164,166] have considered tri- and tetrapeptides. They used various physicochemical properties of individual amino acids and applied them to a large set of peptides to validate the predictive power of their model. Their model has demonstrated a strong relationship of peptide activities to hydrophobic, steric, polarity, and hydrogen bonding properties of individual residues.

The results demonstrate that:

- The properties of the residue next to the C-terminal position play a key role in determining the antioxidant activity of the sequence. For high activity, hydrophilic (Q, N, T, and S) and hydrogen bonding residues (E, D, H, K, D, P, Y, C, and R) are favoured in this position. Specifically, it has been demonstrated that hydrophobic residues are not suitable for this position.
- N-terminal hydrophobic residues (A, V, L) and C-terminal polar residues (W, E, Y, Q) are favourable for potent antioxidant peptides.

The model proposes that the polar residues next to the C-terminus make the peptide a good hydrogen atom donor and easily quench free radicals [164]. For instance, the prawn-derived antioxidant peptides IKK and FKK, and other food-derived antioxidant peptides, satisfy these predicted structural requirements (Table 4). However, further investigations are required to extend the predictive ability of the model to larger peptides and to evaluate the possible role of secondary structures of antioxidant peptides. For example, some food-derived and natural antioxidant peptides (Table 4) do not completely fulfil the requirements predicted by the model due to:

- The limitation of the model that only tri- and tetrapeptides are considered.
- The possible relationship of the antioxidant activities to the secondary structures of peptides.

### 6.4. Anticancer Peptides (ACPs)

Although, significant advancement has already been made in cancer therapy, new treatment options are still required to minimize the undesirable side effects caused by existing therapies [221]. This section explores natural peptides with potent anticancer activities and evaluates their structure–activity relationship. As discussed before, nature provides many bioactive peptides to various life forms as part of their survival mechanism. Scorpions, snakes, jelly fish, spiders, and many other animals/organisms have evolved for millions of years and employ their venom for self-defence [221]. These venoms contain a multitude of bioactive peptides that display a spectrum of activities including anticancer activity [27,221]. This subsection focusses on venom-derived anticancer peptides (ACPs). Particular attention is given to scorpion- and snake-derived anticancer peptides [27,221] as there is a significant quantity of literature in this area.

Several potent scorpion-venom-derived ACPs are presented in Table 5. The majority of these peptides display selectivity towards cancer cells with minimal toxicity against normal cells [222,223,224], which is an important factor for further investigations. Generally, these ACPs are cationic peptides that have a propensity to display an α-helical structure in the membrane-mimicking environment and their sequences contain a significant number of hydrophobic residues [223]. The mode of action of these ACPs involves membrane pore formation and endocytosis that results in membrane defects leading to cell necrosis, cell cycle arrest, and apoptosis [27]. It is interesting to note that the mode of action of ACPs is very similar to that of cationic AMPs illustrated in Figure 3. It should be noted that a few ACPs with short sequences do not display α-helical structures [222]. Possibly their structures are stabilized by disulfide bridges formed by cysteine residues. To date, their mode of action is not known [27,222].

A few cationic peptides derived from snake venom display potent anticancer activity [221,225,226,227]. Some examples of mature snake-venom-derived cathelicidines (CATHs) with anticancer properties include (i) Bf-CATH30 with 30 residues, (ii) Cdt-CATH with 34 residues, and (iii) ∆Pb-CATH4 with 24 residues (Table 5). These cationic peptides contain a large number of hydrophobic residues and display a random coil structure in aqueous solution but acquire an α-helical structure in the membrane-mimicking environment [221,225]. The anticancer action of Bf-CATH results from membrane permeation, DNA binding, and the inhibition of VEGF gene expression, and also the inhibition of key intercellular pathways, leading to cancer cell death [221,225]. In addition to direct anticancer activity, Cdt-CATH displayed immunomodulatory activity that is likely to enhance the efficacy of its anticancer activity [221]. It is interesting to note that these cationic ACPs act with an analogous mode of action to cationic AMPs, indicating that the large pool of natural AMPs reported in the literature are important candidates to screen for anticancer activity [1,2,3,4,5,6,7,8,9,10,11,27,221,222,223,224,225,226,227,228,229,230] To date, only a small number of AMPs from the large published pool have been identified as anticancer peptides [221]. Further research in this area is expected to unravel potent ACPs.

Despite the advancements in this area, significant challenges still exist towards the discovery of peptide-based anticancer drugs. Recent developments to search for ideal peptide candidates and the availability of detailed experimental validation protocols are expected to speed up the discovery of effective anticancer therapeutics [221]. The future is bright in this area with many more snake and other venoms still to be investigated.

**Table 5:** Structures of anticancer peptides (ACPs) from natural sources (scorpion and snake venom).

Name	Primary Structure	Source	References
**Naturally occurring peptides**			
Chlorotoxin	MCMPCFTTDHQMARKCDDCCGGKGRGKCYGPQCLCR	Scorpion venom *(L. quinqestriatus)*	[27,222]
BmKn2	FIGAIARLLSKIF	Scorpion venom *(M. martensii)*	[27,228]
Pantinin-2 (P2)	IFGAIWKGISSLL	Scorpion venom (*P. imperator)*	[27,223]
Pantanin-3 (P3)	FLSTIWNGIKSLL	Scorpion venom (*P. imperator*)	[27,223]
RK1	IDCSKVNLTAECSS	Scorpion venom *(B. occitanus tunetanus)*	[27,224]
TsAP1	FLSLIPSLVGGSISAFK	Brazilian yellow scorpion (*T. serrulatus)*	[27,229]
TsAP2	FLGMIPGLIGGLISAFK	Brazilian yellow scorpion *(T. serrulatus)*	[27,229]
Smp24	IWSFLIKAATKLLPSLFGGGKKDS	Scorpion venom *(S. Maurus palmatus)*	[27,230]
Bf-CATH30	KFFRKLKKSVKKRAKEFFKKPRVIGVSIPF	Snake venom *(Bungarus fasciatus)*	[225]
Cdt-CATH	KRFKKFFKKVKKSVKKRLKKIFKKPMVIGVTIPF	Snake venom *(Crotalus durissus terrificus)*	[226]
∆Pb-CATH4	TRSRWRRFIRGAGRFARRYGWRIA	Snake venom *(Python bivittatus)*	[227]

### 6.5. Antiparasitic Peptides (APPs)

Many developing countries are still affected by endemic diseases caused by parasites such as *Trypanosoma species*, *Leishmania species*, and *Plasmodium species* [231]. Malaria is one of the neglected diseases caused by the *Plasmodium species* that kills a large number of patients worldwide due to lack of effective treatment [231]. Other important parasitic diseases that lack effective treatment protocols include Changas disease and Leishmaniasis. The literature indicates that the peptides isolated from mosquitos and other hosts act as antiparasitic peptides (APPs) [231,232,233,234]. Although the research in this area is still in its infancy, insect peptides present a great potential for the development of therapeutics for parasitic diseases [231].

Some of the antiparasitic peptide candidates reported in the literature are presented in Table 6. Even though malaria is the most researched parasitic disease, there are very limited anti-malarial peptide candidates to date [231,235,236]. Studies involving structure–activity relationships indicate that insect-derived peptides with an α-helical structure and without Cys residues display anti-malarial activity [231,236]. These are the cationic peptides with Lys and Arg residues contributing to their positive charges. Another characteristic of these anti-malarial peptides is the presence of a large number of hydrophobic residues in the N-terminal [185,231,237,238]. Their action initiates the pore formation of the lipid membrane leading to parasitic cell death [231,239]. It is important to note that the mode of action of APPs is remarkably similar to the action of anti-bacterial peptides with minor variations [231,240]. Further research is necessary to discover peptide drug leads and therapeutics to treat these long-neglected parasitic diseases.

It is important to mention here that this review presents some examples of potent bioactive peptides under each activity with a view to relating their structures with corresponding activities. It is not the intention of the authors to list every bioactive peptide published in the literature. Similarly, the structure–activity relationships of the most important activities are discussed in this section. Readers are advised to refer to the published literature cited in this review and important review articles in this field [27,221,231].

## 7. Conclusions

Ever since the first documented use of peptides during World War II, peptide therapeutics have carved a unique niche for themselves and will continue to be important in the pharmaceutical arena. Research into therapeutic peptides continues to expand their use to new molecular targets and indications, broaden their molecular diversity, and improve their pharmaceutical properties. Moreover, new drug delivery systems and formulations and in vivo half-life-extension approaches will further increase the uptake of these distinctive molecules. The pharmaceutical research pipeline is full of peptides that show activity against a vast number of addressable targets with very few approved drugs, showing encouraging results in early-stage and/or preclinical trials. For instance, Kisspeptin analogues targeting GPR54 in assisted reproduction, or agonists of melanocortin 4 receptor (MC4R) for treating genetic obesity syndrome appear to be promising candidates. Other examples include linaclotide and afamelanotide, close analogues of native peptides, targeting guanylyl cyclase C and melanocortin 1 receptor (MC1R), being approved by regulatory agencies for use.

Compared to proteomics, peptidomics is rather an emerging field, wherein continual advancements in screening approaches and computational biology will improve efforts in peptide drug discovery. The currently available peptide drug candidates are not limited to the human peptide pool. Instead, they are primarily sourced from multifarious natural sources and await the characterization of their unique structural features generated due to non-ribosomal synthesis or uncommon post-translational modifications. Bioinformatic approaches focusing on structural studies of peptides, thus, warrant further research. Furthermore, improvement in our understanding of the molecular basis of genetic disorders will also aid in generating and identifying new potential lead peptides, either as biomarkers or therapy options.

## Figures and Tables

**Table 1:** A few classical examples of bioactive peptides from natural sources.

Name	Primary Structure	Source	Activity	Reference
Animal Sources				
	VGINVKCKHSGQCLKPCKDAGMRFGKCINGKCDCTPK	Scorpion venom	Anti-bacterial	[27]
Tachyplesin I	KWCFRVCYRGICYRRCR	Horseshoe crab (*Tachypleus tridentatus*)	Anti-bacterial	[28]
	SIITMTKEAKLPQLWKQIAC-RLYNTC	Yunnan frog, Rana pleuraden	Antioxidant	[29]
Temporin 1Tb (TB)	ALWKTMLKKLGTMALHAGKAALGAAADTISQGTQ	Frog skin	Antimicrobial	[30]
Kappacin	AVESTVATLEDƩPEVIESPPE	Bovine milk	Anti-bacterial	[31]
	IVSDGNGMNAWVAWR	Chicken egg	Anti-bacterial	[32]
	GQGAKDMWR	Donkey milk	Antioxidant	[33]
	EWFTFLKEAGQGAKDMWR	Donkey milk	Antioxidant	[33]
Hydrolysates of camel milk protein	KDLWDDFKGL	Camel milk	Anti-diabetic	[34]
	MPSKPPLL	Camel milk	Anti-diabetic	[34]
	KFQWGY	Camel milk	Inhibition of cholesterol esterase	[35]
	SQDWSFY	Camel milk	Inhibition of cholesterol esterase	[35]
	YWYPPQ	Camel milk	Inhibition of cholesterol esterase	[35]
**Marine Sources**				
	AERQ	Coral (*Sarcophyton glaucum*)	Anticancer	[36]
	RDTQ	Coral (*Sarcophyton glaucum*)	Anticancer	[36]
	AGAPGG	Coral (*Sarcophyton glaucum*)	Anticancer	[36]
	LSGYGP	Tilapia (*O. niloticus*) skin	ACE inhibitory	[37]
	CPAP	*Chlorella pyrenoidosa*	Anticancer	[38]
	VECYGPNRPQF	*Chlorella vulgaris*	Antioxidant	[39]
	ALLAGDPSVLEDR	*Bangia fusco purpurea*	Antihypertensive	[40]
	VVGGTGPVDEWGIAGAR	*Bangia fusco purpurea*	Antihypertensive	[40]
	VKAGFAWTANQQLS	Tuna backbone	Antioxidant	[41]
**Plant Sources**				
	FFL	Soy	ACE inhibitory	[42]
	RQSHFANAQP	Chickpea (*Cicer anetinum*)	Anticancer	[43]
	AIRQGDVF	Crude rice bran	Antioxidant	[44]
	FGER	Potato	Antioxidant	[45]
	DAQEFKR	Kamut (*Triticum turanicum* Jakubz.)	Antioxidant	[46]
	DNIPIVIR	Wheat (*Triticum aestivum* L.)	Antioxidant	[46]
	GNQEKVLELVQR	Spelt (*Triticum spelta* L.)	Antioxidant	[46]

**Table 2:** Summary of extraction approaches for global peptidome analysis.

	Extraction Method	Principle	Pros	Cons	References
**Bottom-Up Approaches**	Enzymatic hydrolysis	Proteolytic cleavage by peptidases at specific sites to yield peptide fragments	(a) Enzyme-specific cleavage sites	(a) Loss of critical structural information of intact peptides and/or truncated proteins	[50]
			(b) Fast process		
	Microbial fermentation	Proteolytic cleavage by microbial peptidases at specific sites to yield peptide fragments	(a) Enzyme-specific cleavage sites	(a) Loss of critical structural information of intact peptides and/or truncated proteins	[26]
				(b) Presence of biological impurities and immunogenicity concerns	
				(c) Differential proteolytic activity expressed by each culture—unpredictability of peptides obtained	
				(d) Long reaction times and difficult to scale up operations	
**Top-Down Approaches**	Ultrafiltration	Molecular weight cut-off: Membrane-based separation allows molecules only under the specified limit to pass through	(a) Simple and cost-effective	(a) Non-specific binding by peptides and clogging membrane pores affect recovery	[50,57,58,59]
			(b) Fast process	(b) Peptides with molecular weights closer to the cut-off may be lost	
				(c) Possible filtrate contamination	
	Organic protein precipitation	Solvent addition causes the precipitation of larger proteins, leaving peptide fragments in the supernatant	(a) Simple, efficient, and cost-effective	(a) The precipitating solvent chosen must be optimized for every biological matrix to ensure maximum peptide extraction	[50,59]
				(b) Entrapment of smaller peptides within large protein aggregates—inconsistencies in extraction yield	
	Acidic protein precipitation	Solvent addition causes the precipitation of larger proteins, leaving peptide fragments in the supernatant	(a) Simple, efficient, and cost-effective	(a) Affected by peptide solubility at acidic pH	[50]
			(b) Rapid protein precipitation process, avoiding protease-related degradation		
	Size exclusion chromatography (SEC)	Chromatographic columns with specific pore sizes exclude and cause early elution of high-molecular-weight biomolecules, while smaller peptides enter the pores and elute out later	(a) Simple and reproducible	(a) High elution volume leading to sample dilution and increased cost—low throughput	[49,50,59]
				(b) Higher sample load requirement	
				(c) Low resolution—needs to be coupled with other separation techniques	
	Solid-phase extraction (SPE)	Peptides of interest interacting with the stationary phase SPE sorbent are retained, while other interfering molecules are washed off with the solvent(s)	(a) Compatible with MS and other high-throughput techniques	(a) Newer versions such as mixed-mode or restricted access matrix sorbents are exceptionally selective and unsuitable for global peptidomics	[50,59,60]
			(b) High resolution	(b) Possibility of slight protein co-elution	
	Combination of solvent precipitation and solid-phase extraction (SPE)	Organic solvent precipitation removes large molecules from the sample, while the subsequent SPE removes the leftover small hydrophobic interfering biomolecules	(a) Highly effective in removing all interfering molecules	(a) Lengthy process with increased extraction steps	[50]

**Table 3:** Structures of ACEIPs from natural resources and food proteins.

Name	Primary Structure *	Source	References
**Food-protein-derived peptides**			
TBS1	IPP	Sour milk	[195]
	VPP	Sour milk	[195]
	AR	Trevally (*Pseudocaranx* sp.)	[65]
	LKP	Egg white	[162,196]
	VNP	Rice	[197]
	VWP	Rice	[197]
	VY	Sake	[198]
	FY	α-zein	[199]
	IY	Sardine	[200]
	AF	Rabbit	[201]
	FFYY	Processed soya milk	[202]
LPI5	EPLYV	Leatherjacket (*Meuchenia* sp.)	[203]
LPI6	DPHI	Leatherjacket (*Meuchenia* sp.)	[203]
LBI5	AER	Leatherjacket (*Meuchenia* sp.)	[203]
**Naturally occurring peptides**			
Bn-PRO-10a	*p*ENWPRPKIPP	*Bitis gabonica rhinoceros* venom	[204]
Bj-PRO-10b	*p*ENWPRPQIPP	*Bothrops jararaca* venom	[192,201,205]
Peptide F	*p*ELWPRPHIPP	*Agkistrodon piscivorus piscivoris* venom	[206,207]
POL 236	*p*ELWPRPQIPP	*Crotalus atrox* snake venom	[208]
Br-PRO-10a	*p*ENWPHPQVPP	*Bitis gabonica rhinoceros* venom	[204]
Bg-PRO-11a	*p*EWQRPGPEIPP	*Bothrops jararaca* venom	[204]
Bn-PRO-10c	*p*ENWPRPKVPP	*Bothrops jararaca* venom	[204]

* *p*E = pyroglutamic acid residue.

**Table 4:** Structures of antioxidative peptides from natural resources and food proteins.

Name	Primary Structure	Source	Reference
**Food-protein-derived peptides**			
	LPHSGY	Alaska pollack (*Theragra chalcogramma*)	[212]
	GSTVPERTHPACPDFN	Hoki (*Johnius belengerii*)	[214]
	PSKYEPFV	Grass carp	[215]
	LHY	Sardinelle (*Sardinella aurita*)	[165]
	VKEAMAPK	Bovine β-casein	[216]
	AVPYPQR	Bovine β-casein	[216]
	YVEEL	Whey proteins	[217]
	TEINEGALLLPH	Lupin seed (*Lupinus angustifolius*)	[218]
	EAGTIETWNPN	Lupin seed (*Lupinus angustifolius*)	[218]
**Naturally occurring peptides**			
Brevinin-1TP2	FLPGLIKAAVGIGSTIFCKISKKC	East Asian frog (*Hylarana taipehensis*)	[219]
Temporin-TP1	FLPVLGKVIKLVGGLL	East Asian frog (*Hylarana taipehensis*)	[219]
Parkerin	GWANTLKNVAGGLCKITGAA	Xizang plateau frog (Nanorana parkeri)	[220]

**Table 6:** Structures of antiparasitic peptides (APPs) from natural sources.

Name	Primary Structure	Insect Source	References
**Naturally occurring peptides**			
Cecropin A	KWKLFKKIEKVGQNIRDGIIKAGPAVAVVGQATQIAK	*H. cecropia*	[231,241]
Cecropin B	KWKVFKKIEKMGRNIRNGIVKAGPAIAVLGEAKAL	*H. cecropia*	[231,242]
Melittin	GIGAVLKVLTTGLPALISWIKRKRQQ	*Apis melífera venom*	[231,232]
Meucine-24	GRGREFMSNLKEKLSGVKEKMKNS	*A. melífera venom*	[231,235]
